# Alteration in the Cerebrospinal Fluid Lipidome in Parkinson’s Disease: A Post-Mortem Pilot Study

**DOI:** 10.3390/biomedicines9050491

**Published:** 2021-04-29

**Authors:** Joaquín Fernández-Irigoyen, Paz Cartas-Cejudo, Marta Iruarrizaga-Lejarreta, Enrique Santamaría

**Affiliations:** 1 Clinical Neuroproteomics Unit, Navarrabiomed, Complejo Hospitalario de Navarra (CHN), Instituto de Investigación Sanitaria de Navarra (IdiSNA), Universidad Pública de Navarra (UPNA), 31008 Pamplona, Spain; jfernani@navarra.es (J.F.-I.); pazcarce@hotmail.com (P.C.-C.); 2 Metabolomics Department, One Way Liver S.L. (OWL), 48160 Derio, Spain; miruarrizaga@owlmetabolomics.com

**Keywords:** lipids, cerebrospinal fluid, Parkinson’s disease, mass-spectrometry, lipidomics

## Abstract

Lipid metabolism is clearly associated to Parkinson’s disease (PD). Although lipid homeostasis has been widely studied in multiple animal and cellular models, as well as in blood derived from PD individuals, the cerebrospinal fluid (CSF) lipidomic profile in PD remains largely unexplored. In this study, we characterized the post-mortem CSF lipidomic imbalance between neurologically intact controls (*n* = 10) and PD subjects (*n* = 20). The combination of dual extraction with ultra-performance liquid chromatography-electrospray ionization quadrupole-time-of-flight mass spectrometry (UPLC-ESI-qToF-MS/MS) allowed for the monitoring of 257 lipid species across all samples. Complementary multivariate and univariate data analysis identified that glycerolipids (mono-, di-, and triacylglycerides), saturated and mono/polyunsaturated fatty acids, primary fatty amides, glycerophospholipids (phosphatidylcholines, phosphatidylethanolamines), sphingolipids (ceramides, sphingomyelins), N-acylethanolamines and sterol lipids (cholesteryl esters, steroids) were significantly increased in the CSF of PD compared to the control group. Interestingly, CSF lipid dyshomeostasis differed depending on neuropathological staging and disease duration. These results, despite the limitation of being obtained in a small population, suggest extensive CSF lipid remodeling in PD, shedding new light on the deployment of CSF lipidomics as a promising tool to identify potential lipid markers as well as discriminatory lipid species between PD and other atypical parkinsonisms.

## 1. Introduction

Due to the lipid heterogeneity, it has been estimated that the human lipidome may be composed by 100,000 different lipid species [1,2]. Lipids play multiple roles in brain function, affecting the elasticity and structural organization of synaptic membranes and modulating protein activity involved in cellular signaling dynamics [3,4,5]. In the context of PD, a genetic risk has been characterized between lipid/lipoproteins traits and the disease [6]. Mutations in lipid-producing enzymes, such as GBA, associated with familial PD and SNPs in multiple PD related-genes involved in lipid homeostasis [7,8,9,10,11] (*SREBF1*, *ASAH1*, *SMPD1*, *PLA2G6*, amongst others) have been linked to PD. Moreover, lipids not only influence in the aggregation potential of alpha-synuclein in vitro and in vivo [12], but they are also present in high concentration as components of crowded membranes, vesicle structures and dysmorphic organelles present in Lewy bodies (LB) [13]. All these data evidence that lipid metabolism should be tightly regulated to counteract the appearance and progression of PD. Systematic studies of cases with LB pathology have prompted a staging classification of PD based on the putative progression with time of LB pathology in the brain from the medulla oblongata and olfactory bulb to the neocortex.

Lipidomics is emerging as a powerful approach that complements protein and gene-centric workflows in the biomarker search to evaluate the neurodegenerative risk or the neurodegenerative progression [14]. Although the scientific community is on a continuous learning curve to obtain a comprehensive portrait of the human brain lipidome [15], the deployment of different variants of chromatographic separations coupled to mass-spectrometry is considered the gold standard approach to study lipid profiles in a high-throughput manner. However, multiple efforts are needed to solve and standardize the associated analytical challenges [16]. Several lipidomic platforms have recently been used to characterize the lipid composition of biofluids in neurological disorders such as amyotrophic lateral sclerosis, multiple sclerosis and Alzheimer’s disease (AD) [17,18,19,20,21]. In this study, we applied a discovery workflow to determine the global lipidomic changes at the CSF level between PD and controls using ultra-performance liquid chromatography-electrospray ionization time-of-flight mass spectrometry (UPLC-ESI-ToF-MS), monitoring more than 250 lipid species and detecting a new metabolic signature associated with the disease that should be further validated in extensive sample cohorts in terms of biomarker sensitivity and specificity.

## 2. Materials and Methods

### 2.1. Materials

Internal standard (IS) compounds, nonadecanoid acid, dehydrocholic acid and tryptophan-(indole-d5), were purchased from Sigma-Aldrich-Merck KGaA (Darmstadt, Germany). 1-tridecanoyl-2-hydroxy-sn-glycero-3-phosphocholine (13:0 Lyso PC), N-hexanoyl-D-erythro-sphingosylphosphorylcholine SM (d18:1/6:0), 1,2-diheptadecanoyl-sn-glycero-3-phosphoethanolamine (17:0 PE), 1,2-dinonadecanoyl-sn-glycero-3-phosphocholine (19:0 PC) and N-heptadecanoyl-D-erythro-sphingosine Ceramide (d18:1/17:0) were purchased from Avanti Polar Lipids (Merck KGaA, Darmstadt, Germany). Tritridecanoylglycerol (13:0 TG), Triheptadecanoylglycerol (17:0 TG) and Cholesteryl Laurate ChoE (12:0) were purchased from Larodan Fine Chemicals (Solna, Sweden). All chemicals and solvents (acetonitrile, methanol, water, isopropanol, formic acid, ammonium formate) were of analytical, HPLC or HPLC-MS grade. See Appendix A for IS working solution preparations (Table A1 and Table A2).

### 2.2. Metabolite Extraction from CSF

Control (*n* = 10; mean age: 77.7 years; 4F/6M) and PD (*n* = 20; mean age: 79.9 years; 7F/13M) post-mortem CSF samples were obtained from the Parkinson’s UK Brain Bank funded by Parkinson’s UK, a charity registered in England and Wales (258197) and in Scotland (SC037554) (Table 1). During the post-mortem brain removal (PMI < 24 h.), the CSF was obtained as follows. The tentorium cerebelli was cut close to its attachment to the skull base (on the petrous bone). CSF was obtained anteriorly to the brainstem. After a centrifugation step (3 min at 10,000 rpm), CSF aliquots were frozen at −80 °C. Metabolite extraction was performed as previously described [22]. Briefly, 150 μL of CSF was spiked with 600 μL and 570 μL of ice-cold IS working solution for lipidomics platforms 1 and 2, respectively. Once spiked with the extraction solvents, samples were mixed with 570 μL of ice-cold CHCl3, vortexed for 20 min at RT and incubated for 1 h at 4 °C. Then, a centrifugation step was carried out (18,000× *g*, 15 min, 4 °C) and 650 µL of supernatant were collected for each platform. Lipidomics platform 1:650 μL of supernatant were dried at 40 °C in a vacuum concentrator and reconstituted in 50 μL methanol with agitation for 20 min at RT. After centrifugation (18,000× *g* for 5 min at 4 °C) to precipitate any particles, supernatants were transferred to a plate for UPLC^®^-MS analysis. Lipidomics platform 2:650 μL supernatant were mixed with 50 μL of H_2_O and vortexed for a few seconds. After centrifugation (18,000× *g* for 5 min at 4 °C), 400 μL of the lower organic phase were dried at 40 °C in a vacuum concentrator. Dried samples were reconstituted in 50 μL of acetonitrile:isopropanol 1:1 and shaken vigorously at RT for 10 min. A centrifugation step (18,000× *g* for 5 min at 4 °C) was performed to precipitate any particles, and supernatants were transferred to a plate for ultra-performance liquid chromatography UPLC^®^-MS analysis.

### 2.3. Chromatography and Mass-Spectrometry

Lipidomic profiling was carried out by OWL Metabolomics S.L. (Derio, Spain). Briefly, chromatographic separation and mass spectrometric detection conditions employed for each UHPLC-ToF-MS-based platform are indicated in Table 1. An Acquity-LCT Premier XE system and an Acquity-Xevo G2QTOF (Waters Corp., Milford, MA) were used as platform 1 and 2, respectively.

### 2.4. Data Processing and Normalization

TargetLynx application manager for MassLynx 4.1 software (Waters Corp. Milford, MA, USA) was used for data processing. A set of parameters associated to metabolites included in the analysis (Rt *m*/*z*, mass-to-charge ratio pairs, retention time) were incorporated into the program. Using a mass tolerance window of 0.05 Da and after peak detection and noise reduction (at LC and MS levels), only true metabolite related features were processed by the software. For each sample injection, a list of chromatographic peak areas was generated. Data normalization was performed following the procedure described by Barr et al. [23], where the ion intensity corresponding to each peak present in each CSF sample was normalized in respect to the sum of peak intensities in each CSF sample. There were no significant differences (*t*-test = 0.1031) between the total intensities used for normalization of the sample groups compared in the study.

### 2.5. Data Analysis

Once normalized, the dimensionality of the complex data set was reduced to enable easy visualization of any metabolic clustering of the different groups of samples. This was achieved by multivariate data analysis, including the non-supervised principal components analysis (PCA) and/or supervised orthogonal partial least-squares to latent structures (OPLS) approaches. Univariate statistical analyses were also performed, calculating group percentage changes and unpaired Student’s *t*-test *p*-value (or Welch’s *t*-test where unequal variances were found) for the comparison between both experimental groups. To help in the interpretation of lipid changes in a biologically meaningful context, OWLStatApp was used (http://rstudio.owlmetabolomics.com:8031/OwlStatApp).

## 3. Results

During a neurodegenerative process, different types of molecules could be released and finally diffused into the CSF circuit, being considered as potential cerebrospinal fluid (CSF) biomarkers. Because cell membrane breakdown is a characteristic feature of a neurodegenerative process in brain syndromes, the deep characterization of CSF metabolomic profiles could reveal specific lipid molecules released by damaged neuronal or glial cell populations, establishing novel molecular panels to help us in the characterization of neurodegenerative diseases. In the current study, we have focused our attention on the metabolic profile of CSF lipids in PD.

### 3.1. Categorization of the Detected CSF Lipidome

**Figure 1:**
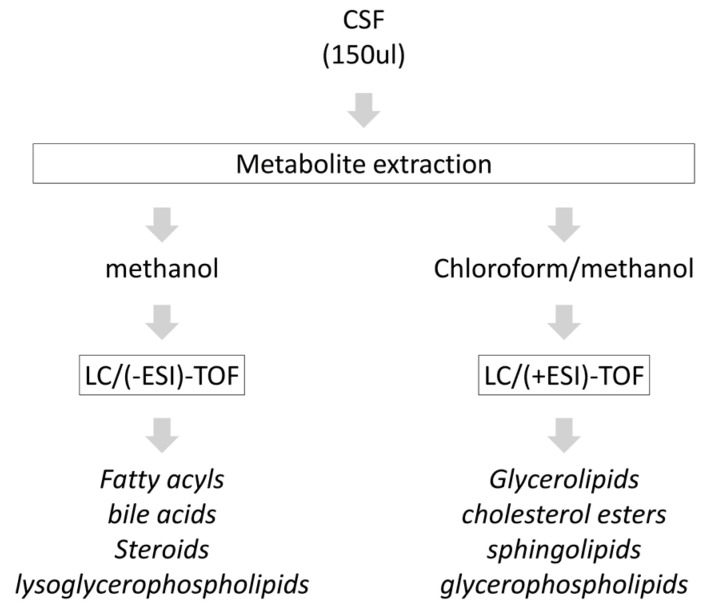
Lipidomic workflow applied in our pilot study.

Due to the wide concentration range of lipids and their extensive chemical diversity [1], it is not possible to analyze the full lipidomic profile in a single experiment. Therefore, lipid extraction was carried out by fractionating the post-mortem CSF samples into groups of species with similar physicochemical properties, using appropriate solutions of organic solvents (methanol, chloroform/methanol) and then analyzing the different extracts in specific analytical platforms [23]. In our case, two UHPLC-MS based platforms were used (Figure 1) to maximize the analysis of CSF lipidomic profiles derived from neurologically intact controls and PD subjects (Table 2), performing an optimal profiling of: (i) fatty acyls, bile acids, steroids and lysoglycerophospholipids; and (ii) glycerolipids, glycerophospholipids, sterol lipids and sphingolipids. Using this dual workflow, a total of 257 metabolic features were detected in all human CSF samples, including 6 bile acids, 10 fatty amides, 3 acylcarnitines, 65 glycerolipids, 111 glycerophospholipids, 22 non-esterified fatty acids, 33 sphingolipids and 7 sterols (Table S1).

### 3.2. CSF Lipidomic Profiling in Parkinson’s Disease

The 257 detected lipid features were analyzed across all CSF samples. Once normalized, the dimensionality of the complex dataset was reduced to enable easy visualisation of any metabolic clustering of the PD and control CSFs. The quality of the global experiment was assessed (see Appendix A).

#### 3.2.1. Multivariate Analysis

**Figure 2:**
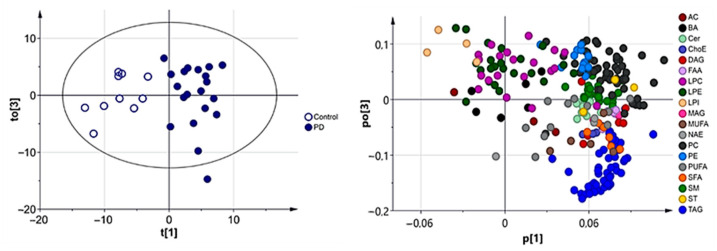
Score scatter plot (**left panel**) and loadings scatter plot (**right panel**) of the OPLS-DA model of CSF samples after square root transformation of the data. Model diagnostics (A = 9; R2X = 0.860; Q2X = 0.150).

**Figure A4:**
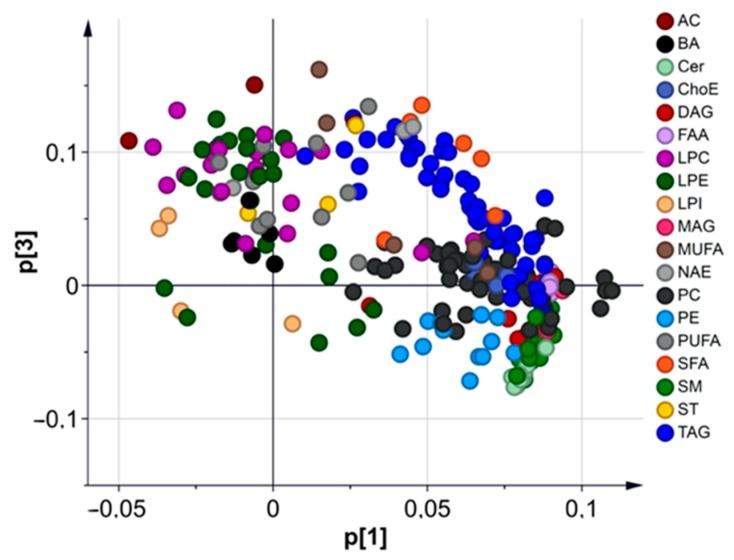
Loadings scatter plot of the PCA model of cerebrospinal fluid samples after square root transformation of the data. Model diagnostics (A = 4, R2X = 0.752, Q2X = 0.505).

A supervised OPLS model was also calculated in order to achieve the maximum separation between both experimental groups. Figure 2 (left panel) shows the score scatter plot of this model, in which a clear clustering of CSF samples according to the presence or absence of PD was observed. Similar to what was found for the loadings scatter plot displayed in Figure A4 (Appendix A), metabolites responsible for the differences observed were mainly glycerolipids (MAG, DAG, TAG), fatty acids (SFA, MUFA), FAA, glycerophospholipids (PC, PE) and sphingolipids (Cer, SM), which were increased in the PD group (Figure 2, right panel). However, this model had a low predictive ability (Q2X = 0.150), indicating it would be necessary to extend this pilot study to include additional sample cohorts.

#### 3.2.2. Univariate Analysis

**Figure 3:**
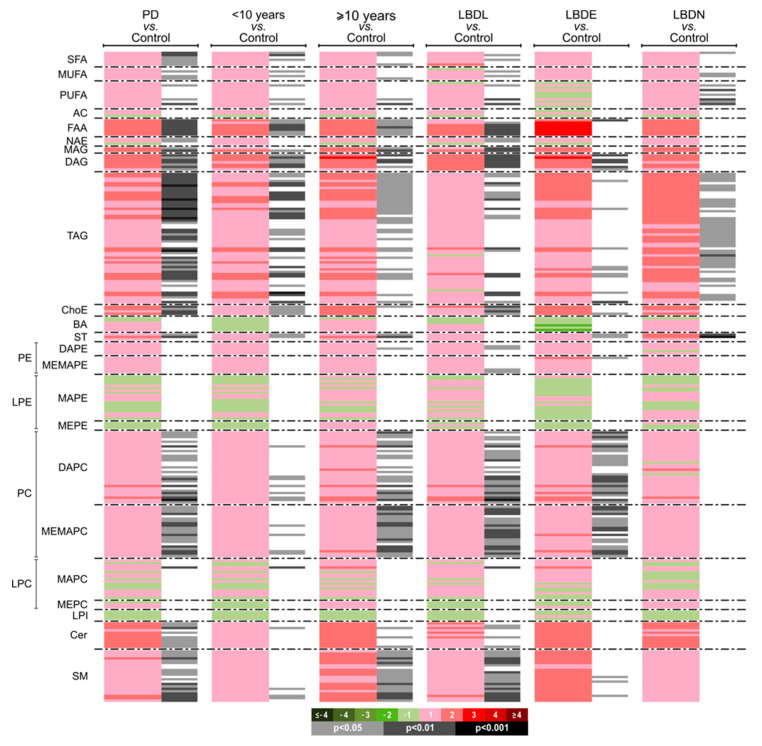
Heatmap representing differential individual metabolic features obtained from the global PD and control comparison and based on disease duration and neuropathological staging. Log transformed ion abundance ratios are depicted, as represented by the scale. Darker green and red colors indicate the change intensity of the metabolite levels, respectively. Grey lines correspond to significant fold-changes of individual metabolites; darker grey colors have been used to highlight higher significances (*p* < 0.05, *p* < 0.01 or *p* < 0.001). It is relevant to highlight that metabolites have been ordered in the heatmap according to their carbon number and unsaturation degree of their acyl chains. Heatmap color codes for log2 (fold change) and unpaired Student’s *t*-test *p*-values are indicated at the bottom of the heatmap. Metabolite order is supplied in the “Heatmap datasheet” in Tables S1–S3.

Univariate data analysis was also performed, calculating group percentage changes and unpaired Student’s *t*-test *p*-value (or Welch’s *t* test where unequal variances were found) for the PD vs. control comparison. As mentioned in Appendix A, a Shapiro–Wilk test revealed that the majority of the CSF metabolites measured from PD were not following a normal distribution. Then, in addition to the untransformed data analysis, a square root (sqrt) transformation of the data was also applied. Raw intensity data, average group intensities, fold changes and an unpaired Student’s *t*-test of each individual metabolite and of each metabolic class for both untransformed and sqrt transformed data are included in Table S1. In order to correlate the alteration in specific lipid classes with Lewy body disease (LBD) staging, we classified the PD group according to the neuropathological staging: Lewy body disease (LBD) limbic stage (LBDL), LBD early-neocortical stage (LBDE) and LBD neocortical stage (LBDN) (Table 2). Moreover, to deepen our understanding of the lipid-dependent effects on PD duration, an additional analysis was performed, evaluating the correlation between the CSF lipidomic dysregulation and the disease duration in our sample cohort. To obtained balanced subgroups, we divided our PD cohort using a cutoff point of 10 years, generating two groups: (i) <10 years (9 subjects) and (ii) ≥10 years (11 subjects). The raw data per metabolic class was calculated as the sum of the normalized areas of all the metabolites with the same chemical characteristics. In order to help in the visualization of the results, a heatmap was generated. The heatmap in Figure 3 displays the log2 (fold-change) of the 257 metabolites included in all comparative analyses together with the unpaired Student’s *t*-test obtained using the square root (Sqrt) transformation of the data.

According to disease duration, the deregulated lipid classes were highly similar between both groups, except for the phosphatidylcholines and sphingomyelins profiles that were most significantly deregulated in PD subjects with a disease duration of ≥10 years (Tables S2 and S3). According to the neuropathological classification and CSF lipidomic profiles (Tables S2 and S3), primary fatty amides (FAA), cholesteryl esters (ChoE) and sphingomyelins (SM) were most significantly increased in LBDL. A similar phosphatidylcholine profile was significantly elevated in CSF from LBDL and LBDE. However, the CSF lipid profile was reversed in LBDN, where a significant increment was mostly observed at the level of polyunsaturated fatty acids (PUFA) and triacylglycerols (TAG) (Figure 3).

**Figure 4:**
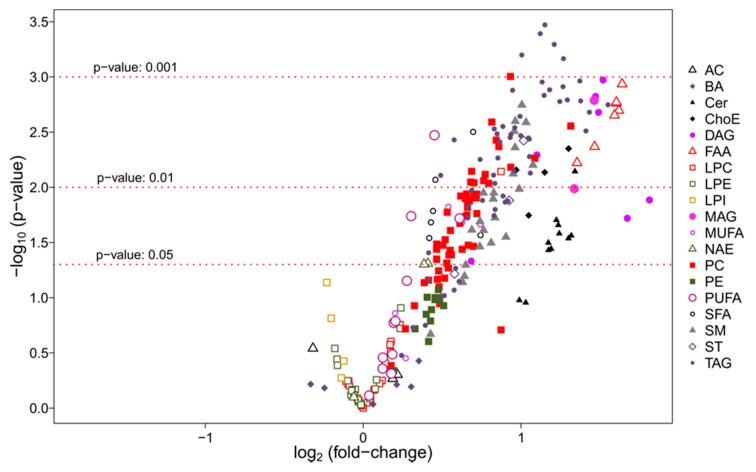
Volcano plot [−log10(*p*-value) vs. log2(fold-change)] for the PD vs. control subjects comparison. This volcano plot highlights the significance *p*-value < 0.01 for glycerolipids and, more specifically, triacylglycerols (TAG).

A volcano plot was generated highlighting the most significant metabolites considered individually for the PD vs. control comparison (Figure 4).

**Figure 5:**
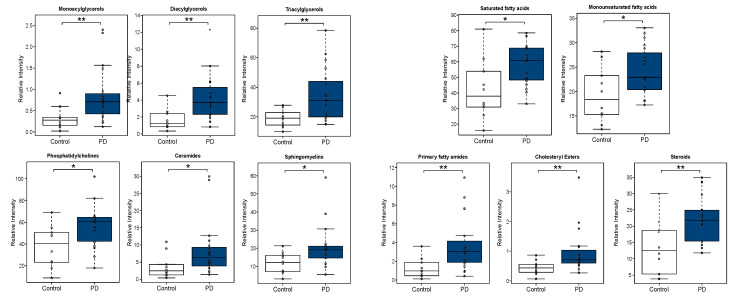
Boxplots of glycerolipids (monoacylglycerols (MAG), diacylglycerols (DAG), triacylglycerols (TAG)), phosphatidylcholines (PC) and sphingolipids (ceramides (Cer), sphingomyelins (SM)) (**left**). Boxplots of non-esterified fatty acids (NEFA) (saturated fatty acids (SFA), monounsaturated fatty acids (MUFA)), primary fatty amides (FAA) and sterol lipids (cholesteryl esters (ChoE), steroids (ST)) (**right**). significances (*; *p* < 0.05 and **; *p* < 0.01).

Lipid classes were also calculated as the sum of the normalized areas of all the lipid metabolites with the same chemical characteristics (Table S1). Interestingly, all lipid classes significantly altered in PD subjects were increased. Changes in some of the most relevant metabolite classes are depicted in the boxplots shown in Figure 5.

**Figure 6:**
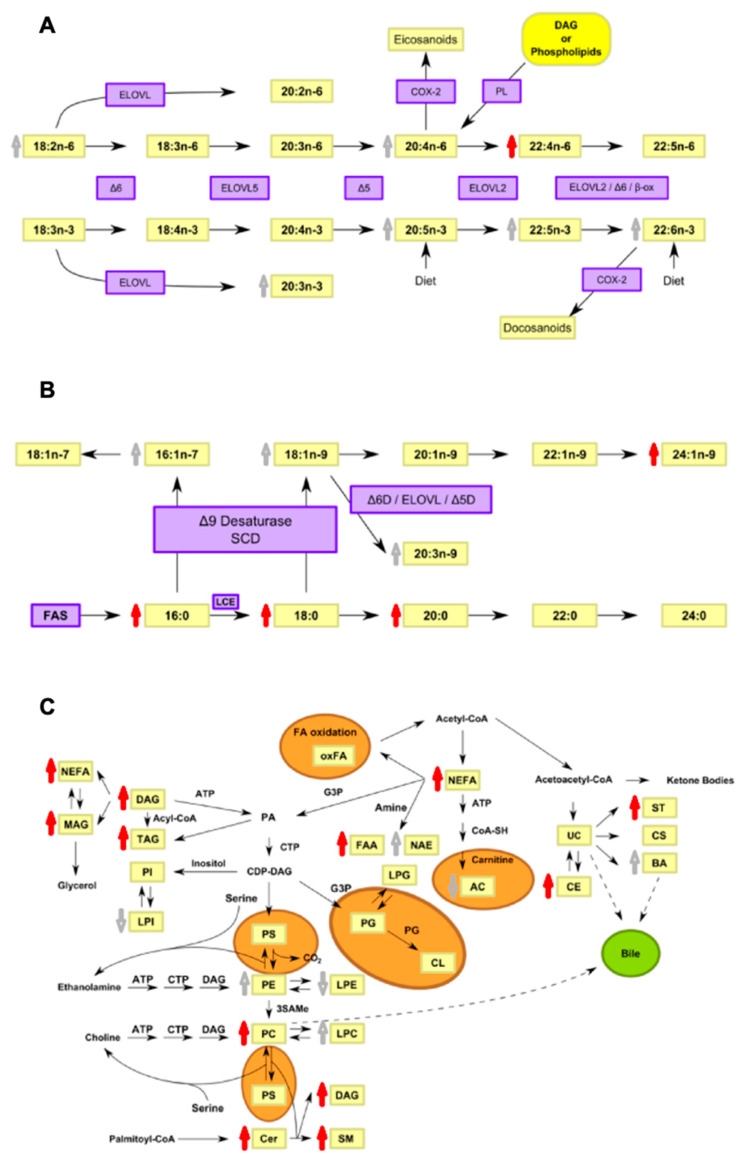
Pathway localization of deregulated lipid species detected in post-mortem CSF in PD. (**A**) Biosynthetic pathway of n-3 and n-6 fatty acids, (**B**) de novo lipogenesis and (**C**) lipid biosynthesis. Delta-6 desaturase (Δ6D), Delta-5 desaturase (Δ5D), elongase (ELOVL), beta-oxidation (β-ox), cyclooxygenase-2 (COX-2), phospholipases (PL). Fatty acid synthase (FAS), long-chain elongase (LCE), stearoyl-CoA desaturase (SCD), Glycerol 3-phosphate (G3P), phosphatidic acids (PA), phosphatidylinositols (PI), lysophosphatidylinositols (LPI), acyl carnitines (AC), unesterified cholesterol (UC), cholesterol sulfate (CS), cholesteryl esters (CE), steroids (ST), phodphatidylserines (PS), phosphatidylglycerols (PG), lysophosphatidylglycerols (LPG), cardiolipins (CL), S-adenosylmethionine (SAMe). Red arrows indicate significant increments in CSF lipid levels (*p* < 0.05). Grey arrows indicate non-significant increments in CSF lipid levels (*p* > 0.05). Orange areas represent routes carried out at the mitochondrial level.

In order to help in the interpretation of the potential origin of the lipidomic changes in a biologically meaningful context, pathway analysis was performed, mapping the deregulated lipid species as well as the lipid metabolic enzymes involved in lipid biosynthetic routes (Figure 6).

## 4. Discussion

Brain lipids act as the major source of energy, provide insulation to cells and structural integrity to membranes and can be rapidly converted to signaling molecules or to inflammatory intermediates [24]. Thus, changes in lipid metabolism and its reflection on CSF lipid content might have a significant impact on brain function, contributing to PD pathogenesis [11,25]. Although the exact role of lipids in PD is not totally understood, the effects and/or levels of a subset of the lipidome have been partially characterized in plasma as well as in animal/cellular PD models [26]. However, brain levels of lipids may not correlate with plasma levels, so additional CSF measurements are needed to address the gap in knowledge about the potential pathological or compensatory composition of the brain lipidome in PD. In our case, all the lipid species which were found to be significantly increased in parkinsonian post-mortem CSF were: (i) several non-esterified fatty acids (NEFA), including the complete profile of saturated fatty acids (SFA), some monounsaturated fatty acids (MUFA) and a few polyunsaturated fatty acids (PUFA); (ii) various primary fatty amides (FAA) and N-acyl ethanolamines (NAE); (iii) almost the complete profile of glycerolipids, including monoacylglycerols (MAG), diacylglycerols (DAG) and triacylglycerols (TAG), (iv) several cholesteryl esters (ChoE) and steroids (ST), (v) almost the complete profile of phosphatidylcholines (PC) and (vi) the majority of ceramides (Cer) and sphingomyelins (SM). Moreover, our study demonstrated that CSF lipid homeostasis is differentially disrupted depending on neuropathological staging and disease duration. Several reasons may explain the over-representation of the characterized CSF lipidome in PD subjects. Lipid dyshomeostasis may be due to extensive synaptic dysfunction, severe lipid raft rearrangements and neuronal death, accompanied by membrane instability and tangled breakdown, contributing to an increment in lipid products in the CSF from PD subjects. Moreover, blood-brain-barrier (BBB) dysfunction is present in PD [27]. The opening of the BBB and the concomitant serum molecular infiltration inside the brain may trigger a multifactorial metabolic imbalance, leading to synaptic and neuronal dysfunction and adverse neuroinflammatory changes. One of these events may be the increment in lipid exchanges between CSF and the blood. However, bearing in mind that our workflow has allowed us to exclusively monitor around 250 lipid species, we cannot exclude the possibility that multiple lipid species not detected in this study may be underrepresented in parkinsonian CSFs. In fact, levels of some bile acids and multiple glycerophospholipids present a non-significant tendency to be lower in PD in respect to the control group (Table S1). Additional studies applying complementary lipidomic strategies in additional patient cohorts will facilitate the global interpretation about the lipid dyshomeostasis across biofluids in PD.

It has been speculated that SFA could exacerbate PD pathology [28]. Moreover, higher SFA levels are present in frontal cortical lipid rafts from PD subjects in respect to controls [29]. Using Drosophila mutant models, it has been shown that alpha-synuclein aggregation is facilitated by phospholipids with shorter acyl chains [30]. Interestingly, saturated phospholipids have been reported to improve alpha-synuclein aggregation and PD-like symptoms [31,32]. Although different CSF MUFA levels have been detected between several PD phenotypes, MUFA levels remain unchanged in the temporal cortex from PD subjects [33,34]. PUFA levels in the anterior cingulate cortex are increased in PD, although their CSF levels depend on the disease etiology [34,35]. At the molecular level, PUFA and alpha-synuclein are involved in the synaptic vesicle cycle [36]. Moreover, it has been evidenced that PUFA increase alpha synuclein oligomerization through the interaction with the N-terminal region [37,38]. With respect to glycerolipids, the exact function of MAG is unknown. While DAG is a secondary lipid messenger that plays a role in the synaptic vesicle cycle [39,40], TAG is directly involved in energy storage [41]. In the context of PD, plasma DAG and TAG tend to be diminished in PD, and higher serum TAG have been linked to a reduced risk of PD [42,43,44]. Alpha-synuclein overexpression has been directly related with intracellular TAG deposition [45,46].

In spite of CSF alterations in several cholesteryl esters (ChoE) and steroids (ST), little is known about the impact of sterols in PD pathogenesis. In general, sterols are known to play a role in immunity, membrane fluidity and serve as signaling mediators [47,48]. In PD, the cholesterol esterifying activity is reduced in fibroblasts and specific ChoE are reduced in the visual cortex [49,50]. Based on data obtained using several PD-related biological systems, it is not evident whether modulation of specific ChoE metabolic events may have a protective or pathological impact [51,52]. Phosphatidylcholine (PC), the most abundant glycerophospholipid in membranes, is involved in the control of inflammation, neuronal differentiation and cholesterol homeostasis [53,54,55]. Our data identified an increment in almost the complete profile of PCs at the level of CSF derived from PD subjects. However, decreased levels in multiple PCs have been observed in plasma, the frontal cortex and substantia nigra from PD patients [42,56,57]. This tendency has been also observed in substantia nigra and brain tissue derived from a mouse model of PD and from MPTP-treated goldfish, respectively [58,59]. Moreover, specific alpha-synuclein isoforms differentially interact with PC membranes [60,61,62,63].

Chronic neuroinflammation is a landmark of PD [64]. Sphingolipids, significantly increased in our study, have been recently proposed as potential diagnostic and therapeutic targets in PD, due to their direct involvement in neuroinflammation [65]. They are particularly relevant in immune-cell trafficking, cytokine signaling, production of pro-inflammatory eicosanoids and the regulation of cellular mechanisms involved in multiple inflammatory processes [66,67]. Specifically, an elevation in ceramide concentration can trigger neuronal apoptosis as well as astrocyte activation, playing a pro-inflammatory role [68,69]. However, the acyl chain length and the cell-type determine the functionality of ceramides as being long-chain ceramide mediators of pro-inflammatory phenotypes in microglial cells, whereas short-chain ones trigger anti-inflammatory mechanisms [65]. It has been proposed that different variations in ceramide (Cer) levels across brain areas may be linked to alpha-synuclein accumulation [26]. However, controversial data exist about the Cer plasma levels in PD patients [42,70,71]. In general, an increment in Cer levels is commonly observed in different studies performed in PD animal and cellular models [72,73,74,75]. However, the consequences associated with Cer increments are not fully understood, being potentially detrimental or beneficial for different PD-related mechanisms. It is important to note that CSF ceramides are also increased in other neurodegenerative diseases, such as AD and ALS, indicating that lipid imbalance may be partially common across neurological disorders [18,76]. Sphingomyelin (SM), a major myelin component, is considered a source of bioactive lipidic molecules which play a role in inflammation, autophagy and cell death [77,78,79,80]. According to our data obtained in CSF, SM accumulation has also been observed in: (i) LB aggregates [81], (ii) the primary visual cortex from PD subjects as well as in substantia nigra from males with PD [49,57] and (iii) PD patients with sphingomyelinase-1 mutations (risk factor) [82,83]. Although multiple factors suggest a potential role of SM accumulation in PD-associated neurodegeneration, more experimental evidence is needed to further elucidate the concise function of SM, not only in alpha-synuclein aggregation, but also in inflammatory balance.

In addition to the CSF Cer increment detected in PD, an increment in specific N-acylethanolamines and primary fatty amides (FAA) were also observed. N-acylethanolamines play an important role in various processes, from anti-inflammatory activities [84,85] to neuroprotective actions in PD models [86,87]. Specific FAA are sleep-inducing factors that may also affect memory processes, depress locomotor activity and are anti-inflammatory, anxiolytic and neuroprotective [88,89,90,91]. Interestingly, increased plasma FAA is associated with CSF beta-amyloids and clinical features [92]. The increment we observed in CSF FAA in PD was probably due to a dysfunctional synthesis-degradation efflux or transport. The major degradative step for FAA is the fatty acid amide hydrolase (FAAH) that degrades FAA to fatty acids and ammonia and also hydrolases the endocannabinoids. Pharmacological inhibition of FAAH leads to the inhibition of dopamine neuron death and reduces the immunoreactivity of microglial cells [93]. However, the precise role of FAA in alpha-synucleinopathies remains to be elucidated. Other lipid species with a pro-inflammatory role, such as platelet activating factors (PAFs) [94] or specific glycosphingolipids related to IL-1beta/IL-18 production, auto-antibody production and recruitment of peripheral immune cells within the CNS [95], were detected in our study, suggesting that additional workflows are needed to elucidate the full picture of inflammation-related CSF lipidome involved in PD.

It is important to note that our data obtained at the level of CSF partially corroborate previous associations between PD and the levels of fatty acyls, glycerolipids, glycerophospholipids, sphingolipids and sterols. Moreover, our pilot study established novel links between primary fatty amides (FAA) and N-acyl ethanolamines (NAE) with PD. However, although our untargeted lipidomic work has uncovered many intricacies in the CSF lipidomic homeostasis in the context of PD, there are potential limitations of our study that warrant discussion. First, due to the technological approach used, we failed to accurately monitor many lipid species present at low levels that might also participate in PD pathophysiology. Second, and based on the current knowledge, it is unclear whether the CSF lipid imbalance observed reflected pathological or compensatory mechanisms. Third, our study did not consider the effect of variables such as sex, age, PD etiology and/or mutational profiles.

Comparing our data with previously published works using early clinical PD biofluid samples, alteration of glycerophospholipid and sphingolipid metabolism was also observed at the plasma level [96]. However, the specific phosphatidylcholine, phosphatidylethanolamine and sphingomyelin profiles clearly differed with respect to our postmortem CSF data. Moreover, several studies have also indicated that sphingolipids (ceramides and sphingomyelins) are elevated in CSF derived from AD patients in respect to cognitively normal individuals [76,97]. Wood PL et al. [98] performed a lipidomic analysis in post-mortem CSF derived from AD subjects. In contrast, the differential lipidomic profile obtained was clearly different with respect to the lipid alterations we observed in post-mortem CSF from PD. Based on these data and taking into account the biomarker field, large cohorts of paired antemortem CSF and plasma samples should be used, not only from PD patients, but also from other synucleinopathies and tauopathies to obtain robust lipid-based conclusions in terms of biomarker specificity and sensitivity.

## 5. Conclusions

A CSF lipidomic approach performed in PD and control subjects (*n* = 30) detected 257 metabolic features by ultra-high performance liquid chromatography-mass spectrometry (UHPLC-MS). A supervised OPLS model showed a clear separation between control and PD subjects, indicating that the lipids responsible for this separation were mainly glycerolipids (MAG, DAG, TAG), fatty acids (SFA, MUFA), primary fatty amides, glycerophospholipids (PC, PE) and sphingolipids (Cer, SM), which were increased in the PD group. Univariate data analysis also revealed a general increase in the CSF lipid metabolic profile in PD. Overall, these results suggest that: (i) multiple CSF lipid species tend to be increased in PD compared to control subjects and (ii) the dyshomeostasis observed in the parkinsonian CSF lipid profile varies depending on the disease duration and the neuropathological staging.

## Figures and Tables

**Table 1:** Chromatographic and mass-spectrometric workflows used in this study.

	Platform 1	Platform 2
Column type	UPLC BEH C18, 1.0 × 100 mm, 1.7 μm	UPLC BEH C18, 2.1 × 100 mm, 1.7 μm
Flow rate	0.140 mL/min	0.400 mL/min
Solvent A	0.05% Formic Acid in water	Water:Acetronitrile (2:3) + 10 mM Ammonium Formate
Solvent B	0.05% Formic Acid in acetonitrile	Acetonitrile:Isopropanol (1:9) + 10 mM Ammonium Formate
(%B), time	0%, 0 min	40%, 0 min
(%B), time	50%, 2 min	100%, 10 min
(%B), time	100%, 13 min	40%, 15 min
(%B), time	0%, 18 min	40%, 17 min
Column temperature	40 °C	60 °C
Injection volume	2 μL	3 μL
Autosampler temperature	10 °C	10 °C
Source temperature	120 °C	120 °C
Nebulisation N_2_ flow	600 L/hour	1000 L/hour
Nebulisation N_2_ temperature	350 °C	500 °C
Cone N_2_ flow	30 L/hour	30 L/hour
Ionization	ESI −ve	ESI +ve
Capillary voltage	2.8 kV	3.2 kV
Cone voltage	50 V	30 V
Type of data	Centroid	Centroid
Scan time	0.2 s	0.2 s
Acquisition range	50–1000 Da	50–1200 Da

Analysis of fatty acyls, bile acids, steroids and lysoglycerophospholipids was carried out with lipidomic platform 1, and analysis of glycerolipids, cholesterol esters, sphingolipids and glycerophospholipids was performed with lipidomic platform 2. Abbreviation: ESI, Electrospray ionization.

**Table 2:** CSF samples included in the lipidomic study.

SAMPLE.ID	Age	Sex	Onset	Duration	NPD
PD354	88	F	77	11	LBDE
PD423	66	F	53	13	LBDE
PD436	90	M	82	8	LBDE
PD520	80	M	56	24	LBDE
PD530	85	M	77	8	LBDE
PD357	71	M	37	34	LBDN
PD450	66	M	47	19	LBDN
PD495	88	F	78	10	LBDN
PD501	89	F	82	7	LBDN
PD537	84	M	74	9	LBDN
PD550	83	F	77	7	LBDN
PD562	79	M	72	7	LBDN
PD636	84	M	65	20	LBDN
PD295	83	M	67	16	LBDL
PD340	67	M	53	14	LBDL
PD356	86	F	75	9	LBDL
PD541	72	M	66	6	LBDL
PD546	84	F	71	13	LBDL
PD579	76	M	55	21	LBDL
PD591	77	M	68	9	LBDL
C022	65	M			aging-related changes
C023	78	F			aging-related changes
C030	77	M			aging-related changes
C008	93	F			aging-related changes
C015	82	M			possible ischaemia
C026	78	F			minimal leukostasis
C032	88	M			aging-related changes
C054	66	M			mild aging-related changes
C064	63	F			microvascular pathology
C076	87	M			aging-related changes

PD: Parkinson’s disease; C: controls. Duration (years). NPD: neuropathological diagnosis; LBDL: Lewy body disease limbic stage; LBDE: Lewy body disease early-neocortical stage; LBDN: Lewy body disease neocortical stage.

**Figure A1:**
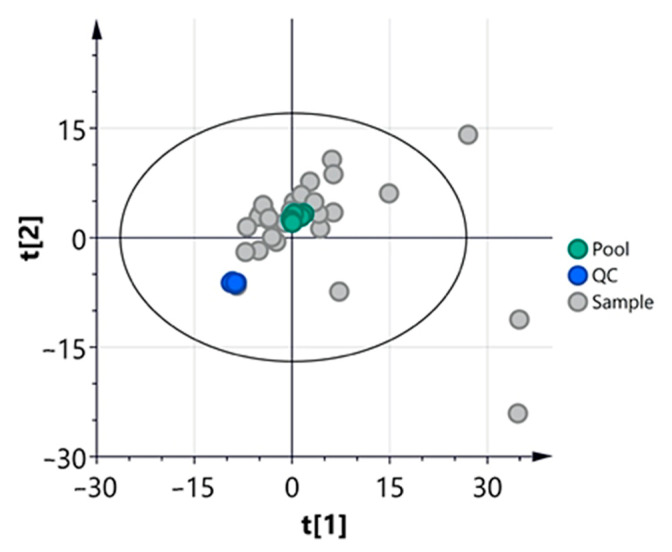
Score scatter plot of the PCA model of CSF, Pool and QC samples. Pool: 15 µl of each CSF sample was collected and pooled together. Model diagnostics (A = 6, R2X = 0.829, Q2X = 0.496).

**Figure A2:**
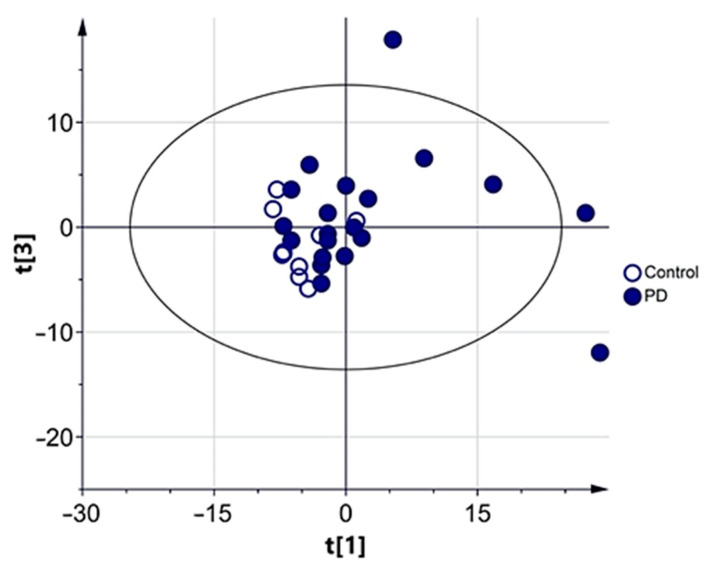
Score scatter plot of the PCA model of CSF samples. Model diagnostics (A = 6, R2X = 0.807, Q2X = 0.401).

**Figure A3:**
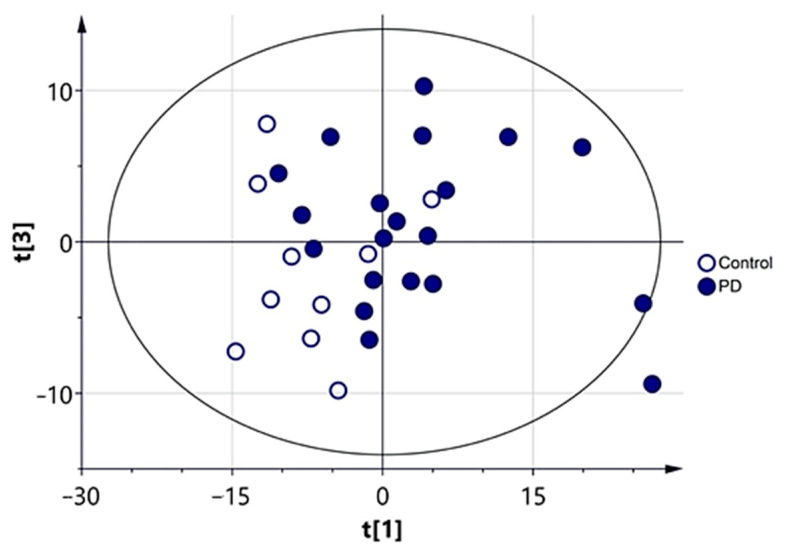
Score scatter plot of the PCA model of CSF samples after square root transformation of the data. Model diagnostics (A = 4, R2X = 0.752, Q2X = 0.505).

## Data Availability

Data available on request from the authors.

## Supplementary Materials

The following are available online at https://www.mdpi.com/article/10.3390/biomedicines9050491/s1, Table S1: “Raw data per metabolite_1” and “Raw data per chemical class_1” contain raw intensity data per metabolites and per metabolite class of the untransformed data, respectively. “Raw data per metabolite_2” and “Raw data per chemical class_2” contain raw intensity data per metabolites and per metabolite class of the data after square root transformation of the data. These sheets also include: (i) “Individual notation”, referring to the confirmed identification of the metabolites. Overlapping of two or more metabolites or non-confirmed identification is indicated in “Individual composition (or probable ID)”, (ii) Average group intensities and standard errors, (iii) Shapiro test: used for testing the normality of data (Shapiro test (*p*) row is marked in red if the sample came from a normally distributed population), (iv) Fold-changes and unpaired Student’s *t*-test *p*-values (or Welch´s *t* test where unequal variances were found) for the comparison PD vs. control and (v) “Heatmap” which contains the metabolites´ identification code, log2(fold-changes) and unpaired Student’s *t*-test *p*-values illustrated in the heatmap. Table S2: “Raw data per metabolite” and “Raw data per chemical class” contain raw intensity data per metabolites and per metabolite class of the untransformed data, considering the neuropathological stage and the disease duration. Table S3: “Raw data per metabolite” and “Raw data per chemical class” contain raw intensity data per metabolites and per metabolite class after square root transformation of the data, considering the neuropathological stage and the disease duration.

## Abbreviations

AC: acyl carnitines; BA: bile acids; Cer: ceramides; ChoE: cholesteryl esters; DAG: diacylglycerides; FAA: fatty acid amides (primary fatty amides); LPE: lysophosphatidylethanolamines; LPI: lysophosphatidylinositols; LPC: lysophosphatidylcholines; MAG: monoacylglycerides; MUFA: monounsaturated fatty acids; NAE: N-acyl ethanolamines; NEFA: non-esterified fatty acids; OPLS: orthogonal partial least-squares to latent structures; PC: phosphatidylcholines; PCA: Principal Component Analysis; PE: phosphatidylethanolamines; PUFA: polyunsaturated fatty acids; SFA: saturated fatty acids; SM: sphingomyelins; TAG: triacylglycerides; UFA: unsaturated fatty acids.

## Appendix A

**Table A1:** Internal Standard (IS) Solutions Platform 1.

IS	IS Stock Solution (µg/mL)	IS Stock Solution	IS Intermediate Solution in CHCl_3_:MeOH (2:1) (µg/mL)	IS Working Solution in MeOH (µg/mL)
**13:0 Lyso PC**	10,000	CHCl_3_	10	0.1
**Dehydrocholic acid**	5000	CHCl_3_:MeOH (1:1)	30	0.3
**Nonadecanoic acid**	10,000	CHCl_3_	500	5.0
**Tryptophan-(indole-d5)**	5000	0.05% Formic acid in water	200	2.0

**Table A2:** Internal Standard Solutions Platform 2.

IS	IS Stock Solution (µg/mL)	IS Stock Solution	Working IS Solution CHCl_3_:MeOH (2:1) (µg/mL)
**SM (d18:1/6:0)**	5000	CHCl_3_	5
**PE (17:0/17:0)**	10,000	CHCl_3_:MeOH:H_2_O	50
**PC (19:0/19:0)**	10,000	CHCl_3_	10
**TG (13:0/13:0/13:0)**	10,000	CHCl_3_	5
**TG (17:0/17:0/17:0)**	10,000	CHCl_3_	5
**Cer(d18:1/17:0)**	10,000	CHCl_3_	10
**ChoE(12:0)**	10,000	CHCl_3_	250

Multivariate data analysis of all CSF samples, pool samples and quality control (QC) samples was initially performed. Score scatter plot corresponding to PCA analysis of these samples is shown in Figure 2. Proximity and overlap of the Pool and QC injections provides a good indication of the reproducibility and quality of the measurements.

After validating the quality of the experiment, the Pool and QC injections were removed from the analysis and a score scatter plot of the PCA model of all cerebrospinal fluid samples was generated.

The Shapiro–Wilk test was used for testing the normality of data (results included in Table S1), revealing that the majority of the metabolites measured in the CSF samples from PD patients were not following a normal distribution. Then, the Box–Cox method for correcting non-normally distributed data by variable transformations was applied, identifying the square root transformation as optimal for most of the metabolites. This kind of transformation is a common pre-treatment method in metabolomics for the conversion of the data, which corrects aspects that hinder the biological interpretation of data sets by emphasizing the biological information and thus, improving their physiological interpretability. Score scatter plot corresponding to PCA analysis of CSF samples after square root transformation of the data is shown in Figure A3.

This score scatter plot showed certain clustering of samples according to the presence or absence of the disease and identified sample PD353 as a potential outlier, since it appeared outside the Hotelling´s T2 ellipse. Following Chauvenet’s criterion, further inspection of the data relating to this sample revealed that it presented elevated levels of sphingolipids compared to the rest of the samples from the PD group. However, the levels of the majority of the metabolites in this sample were similar to those of the samples from the same group and thus, it was not excluded from the multivariate and univariate analyses. Metabolites responsible for this certain separation observed between CSF samples of PD and control subjects can be observed in the loadings scatter plot (Figure A4), which is a graph related to the score scatter plot shown in Figure A3.

Lipids lying away from the plot origin have a stronger impact on the model; besides, variables positively correlated are grouped together, while variables negatively correlated are positioned in the opposite sides of the plot origin. In this case, the metabolites responsible for the differences observed were mainly glycerolipids (monoacylglycerols (MAG), diacylglycerols (DAG), triacylglycerols (TAG)), fatty acids (saturated fatty acids (SFA), monounsaturated fatty acids (MUFA)), primary fatty amides (FAA), glycerophospholipids (phosphatidylcholines (PC), phosphatidylethanolamines (PE)) and sphingolipids (ceramides (Cer), sphingomyelins (SM)), which were increased in PD compared to the control group; and lysoglycerophospholipids (lysophosphatidylcholines (LP), lysophosphatidylethanolamines (LPE), lyshophosphatidylinositols (LPI)) and bile acids (BA), which seemed to be increased in controls compared to the PD group.
